# Myelin Measurement: Comparison Between Simultaneous Tissue Relaxometry, Magnetization Transfer Saturation Index, and T_1_w/T_2_w Ratio Methods

**DOI:** 10.1038/s41598-018-28852-6

**Published:** 2018-07-12

**Authors:** Akifumi Hagiwara, Masaaki Hori, Koji Kamagata, Marcel Warntjes, Daisuke Matsuyoshi, Misaki Nakazawa, Ryo Ueda, Christina Andica, Saori Koshino, Tomoko Maekawa, Ryusuke Irie, Tomohiro Takamura, Kanako Kunishima Kumamaru, Osamu Abe, Shigeki Aoki

**Affiliations:** 1 Department of Radiology, Juntendo University School of Medicine, Tokyo, Japan; 2 Department of Radiology, Graduate School of Medicine, The University of Tokyo, Tokyo, Japan; 3 SyntheticMR AB, Linköping, Sweden; 4 Center for Medical Imaging Science and Visualization (CMIV), Linköping, Sweden; 5 Araya Inc., Tokyo, Japan; 6 Research Institute for Science and Engineering, Waseda University, Waseda, Japan; 7 Research Center for Advanced Science and Technology, The University of Tokyo, Tokyo, Japan; 8 Department of Radiological Sciences, Graduate School of Human Health Sciences, Tokyo Metropolitan University, Tokyo, Japan; 9 Office of Radiation Technology, Keio University Hospital, Tokyo, Japan

## Abstract

Magnetization transfer (MT) imaging has been widely used for estimating myelin content in the brain. Recently, two other approaches, namely simultaneous tissue relaxometry of R_1_ and R_2_ relaxation rates and proton density (SyMRI) and the ratio of T_1_-weighted to T_2_-weighted images (T_1_w/T_2_w ratio), were also proposed as methods for measuring myelin. SyMRI and MT imaging have been reported to correlate well with actual myelin by histology. However, for T_1_w/T_2_w ratio, such evidence is limited. In 20 healthy adults, we examined the correlation between these three methods, using MT saturation index (MT_sat_) for MT imaging. After calibration, white matter (WM) to gray matter (GM) contrast was the highest for SyMRI among these three metrics. Even though SyMRI and MT_sat_ showed strong correlation in the WM (r = 0.72), only weak correlation was found between T_1_w/T_2_w and SyMRI (r = 0.45) or MT_sat_ (r = 0.38) (correlation coefficients significantly different from each other, with *p* values < 0.001). In subcortical and cortical GM, these measurements showed moderate to strong correlations to each other (r = 0.54 to 0.78). In conclusion, the high correlation between SyMRI and MT_sat_ indicates that both methods are similarly suited to measure myelin in the WM, whereas T_1_w/T_2_w ratio may be less optimal.

## Introduction

Myelin is important in the transmission of neural information. It maintains the integrity of neural fibers and enhances the speed of propagation of action potentials, which are essential for the proper function of the brain^1,2^. Measuring myelin in the brain by magnetic resonance imaging (MRI) is important for evaluating the development and aging of healthy humans^3–5^. It is also important for estimating the progression of degenerative^6^ or demyelinating diseases^7^. Conventional MRI is highly sensitive to tissue contrast, but generally unspecific to tissue properties such as myelin content. Furthermore, lengthy scanning time has hindered the routine clinical use of MRI to obtain myelin measurements. Recently, rapid simultaneous relaxometry based on a single pulse sequence has been developed^8^. It quantifies the longitudinal relaxation rate (R_1_), transverse relaxation rate (R_2_), proton density (PD), and local B_1_ field in approximately 6 minutes for full head coverage^9^. The estimated B_1_ field is used for correction of local variations in flip angle. It is possible to create contrast-weighted images (the technique is called ‘synthetic MRI’) such as T_1_-weighted (T_1_w), T_2_-weighted (T_2_w), and fluid-attenuated inversion recovery (FLAIR) images, based on the acquired quantitative values, thus obviating the need for acquiring these contrast-weighted images separately^10^. At the same time, automatic brain segmentation^11^ and myelin measurement^12^ are also possible using the acquired quantitative values. These can be done with a dedicated software called ‘SyMRI’ with post-processing time less than 1 minute^9^. Thus, myelin measurements can now be performed within the limits of clinically allowed scanning time. The myelin model infers myelin volume fraction (MVF) in a voxel based on the effect of myelin on intra- and extracellular water relaxation rates due to magnetization exchange^12^. The observed R_1_ and R_2_ rates of intra- and extracellular water increase in the vicinity of fast relaxing myelin water. On the other hand, the observable PD decreases because myelin water decays much faster than non-myelin water. The SyMRI myelin measurement has been validated on 12 human cadavers using Luxol Fast Blue staining of histological specimens^13^. A repeatability study has reported a very low error (coefficient of variation, 0.59% for 0.8 mm in-plane resolution) for whole-brain myelin volume calculated using SyMRI^14^. Myelin volume measured by SyMRI has been shown to depend on age in pediatric populations, especially in children under 4 years old, thus indicating a correlation of this method with the normal myelination process^15,16^. This method has also been used in studies investigating patients with multiple sclerosis (MS)^17,18^, Sturge-Weber syndrome^19^, and cerebral autosomal dominant arteriopathy with subcortical infarcts and leukoencephalopathy (CADASIL)^20^, with promising results. However, correlation of SyMRI myelin measurement with other MRI techniques sensitive to myelin has not been investigated so far.

There are several other techniques for myelin measurement, including myelin water imaging^21,22^, macromolecular tissue volume derived from normalized PD mapping^23^, and magnetization transfer (MT) imaging^2^. MT is a phenomenon where the proton spins bound to macromolecules, once excited by a radiofrequency pulse, transfer a part of their energy to the neighboring mobile proton spins^24^. MT imaging estimates the macromolecular proton pool size with ultra-short T_2_ relaxation by transfer of magnetization to the observable mobile water pool^25^. MT ratio (MTR) has been widely used based on this theory and shown to correlate well with histological myelin content^26,27^, but also with other properties such as R_1_^24^. R_1_ also correlates strongly with myelin^28^, meaning that MTR and R_1_ work against each other and R_1_ mitigates the power of MTR as a measure of myelin. Further, R_1_ is also sensitive to iron, calcium content, and axon size^29^ and count^30^, thus making the relationship between MTR and actual myelin content nonlinear. MT saturation (MT_sat_) imaging was developed to improve MTR, by decoupling MTR from R_1_^31^. MT_sat_ shows higher contrast in the brain than MTR does^31^, and has been shown to correlate more with disability metrics than MTR in patients with MS^32^. MT_sat_ has also been shown to correlate well with quantitative MT measures^25^, which reduces dependency of MT imaging on sequence parameters. However, quantitative MT imaging is time-consuming and the post-processing is still challenging.

T_1_w/T_2_w ratio is another approach for assessing myelin content in the cortical gray matter, originally developed to map myeloarchitecturally distinct cortical regions for parcellation of cerebral cortex, thus providing a connectivity measurement^33,34^. Pixel intensity on T_1_w and T_2_w images is assumed to be directly and inversely proportional to myelin contrast, respectively. Thus, ratio of these images is thought to accentuate the intrinsic contrast of myelin. Because intensity scaling of T_1_w and T_2_w images differ across scanners and acquisition protocols, Ganzetti *et al*.^35^ have suggested that calibration of their intensities prior to making their ratio can increase the reproducibility of T_1_w/T_2_w ratio. Although T_1_w/T_2_w ratio is not a direct index of myelin, it is still considered a proxy of myelin content^36^. While intracortical myelin content across different ages has been evaluated using this method^36,37^, myelination of white matter (WM) in neonatal brains has also been investigated using this method^38,39^. Further, the test-retest reliability of T_1_w/T_2_w ratio has been reported to be high^40^. Recent histological studies investigated T_1_w/T_2_w ratio in patients with MS, showing that T_1_w/T_2_w ratio was significantly different between myelinated and demyelinated cortex in MS patients^41^, and also significantly different in the cortex between early-stage MS and healthy controls^42^. Because T_1_w and T_2_w images are routinely acquired as part of brain MRI protocols, this technique does not increase scanning time. However, the specificity of T_1w_/T_2w_ to actual myelin content has been doubted by recent studies^40,43^.

As mentioned above, there are several different methods to estimate myelin volume in the brain. However, investigation of correlation among different methods is scarce. Specifically, no study has examined the correlation of SyMRI as a myelin imaging tool with other methods. Therefore, the aim of this study was to compare SyMRI with two other putative myelin measurement techniques by investigating the correlation of SyMRI with MT_sat_ and T_1_w/T_2_w ratio in WM and gray matter (GM).

## Results

### Scatterplots and Mean Values of MVF_MTsat_, MVF_SyMRI_, and MVF_T1w/T2w_

**Figure 1:**
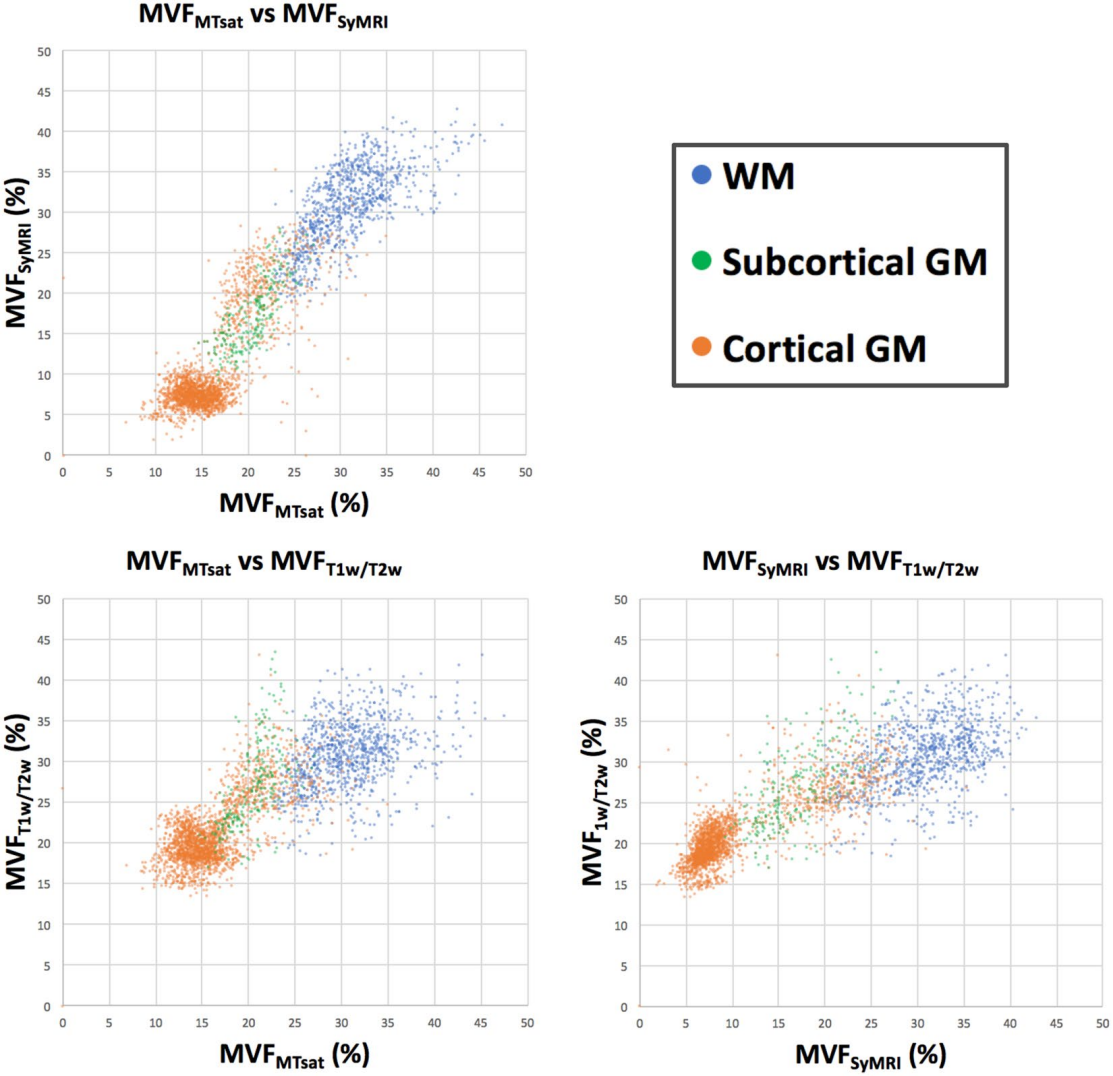
Scatterplots showing correlations among MVF_MTsat_, MVF_SyMRI_, and MVF_T1w/T2w_. For WM, the correlation between MVF_SyMRI_ and MVF_MTsat_ is stronger than the correlation between MVF_T1w/T2w_ and MVF_SyMRI_ or MVF_MTsat_.

The calibration factors for MVF_MTsat_ and MVF_T1w/T2w_ were 8.40 and 14.5, respectively, so that their means in the WM equaled that of MVF_SyMRI_. The scatterplots of these three MVF metrics are shown in Fig. 1. Table 1 shows the mean and standard deviation (SD) of each MVF metric after calibration, and MT_sat_ and T_1_w/T_2_w ratio before calibration in each tissue region, with the percentage of MVF in subcortical or cortical GM to that in WM. Because both MVF_MTsat_ and MVF_T1w/T2w_ were calibrated to MVF_SyMRI_, so that their mean values in the WM were equal, the mean values of WM for all these metrics were the same. The contrasts among WM and subcortical GM, and WM and cortical GM were significantly higher for MVF_SyMRI_ and lower for MVF_T1w/T2w_ than other MVF metrics (*p* < 0.001).

**Table 1:** MVF_MTsat_, MT_sat_, MVF_SyMRI_, MVF_T1w/T2w_, and T_1_w/T_2_w ratio in WM, subcortical GM, and cortical GM, with the percentage of MVF in subcortical or cortical GM to that in WM.

	WM (%)	Subcortical GM (%)	Percentage of MVF in subcortical GM to that in WM (%)	Cortical GM (%)	Percentage of MVF in cortical GM to that in WM (%)
MVF_MTsat_	30.70 ± 4.22	20.55 ± 2.40	66.94*	16.18 ± 3.98	52.70*
MT_sat_	3.66 ± 0.50	2.45 ± 0.29		1.93 ± 0.47	
MVF_SyMRI_	30.70 ± 4.81	17.38 ± 4.11	56.61*	10.57 ± 6.07	34.43*
MVF_T1w/T2w_	30.70 ± 4.03	27.11 ± 5.27	88.31*	21.17 ± 4.15	68.96*
T_1_w/T_2_w ratio	2.11 ± 0.28	1.86 ± 0.36		1.46 ± 0.29	

Data are the mean ± standard deviation.

Note: MVF_MTsat_ and MVF_T1w/T2w_ were calibrated so that their mean in the WM equaled the mean MVF_SyMRI_. *The contrasts among WM and subcortical GM, and WM and cortical GM were significantly different among these three MVF metrics with *p* < 0.001.

### Correlation Coefficients among MVF_MTsat_, MVF_SyMRI_, and MVF_T1w/T2w_

Table 2 shows the Spearman’s ρ correlation coefficients with their 95% confidence intervals (CIs) among MVF metrics. Correlations were significant for all regions—alone or combined—among these metrics (*p* < 0.001). In the WM and subcortical GM, the correlation coefficient was the highest between MVF_MTsat_ and MVF_SyMRI_ (*p* < 0.001 in the WM and *p* = 0.005 in the subcortical GM). In the WM, MVF_T1w/T2w_ showed only weak to moderate correlation with MVF_MTsat_ or MVF_SyMRI_. In the cortical GM, the correlation coefficient was the highest between MVF_SyMRI_ and MVF_T1w/T2w_ (*p* < 0.001), with MVF_MTsat_ vs. MVF_T1w/T2w_ showing the lowest value (*p* = 0.011). In all regions combined, all these metrics showed strong correlations. Correlation coefficients of MVF_MTsat_ vs. MVF_SyMRI_ and MVF_SyMRI_ vs. MVF_T1w/T2w_ were comparable (*p* = 0.62) and higher than that of MVF_MTsat_ vs. MVF_T1w/T2w_ (*p* < 0.001) Table 3 shows the Spearman’s ρ correlation coefficients among MVF metrics in individual areas representative of 10 WM, 2 subcortical GM, and 4 cortical GM, and their mean values. Out of 10 WM ROIs, 8 showed significant correlations between MVF_MTsat_ and MVF_SyMRI_. The 2 WM ROIs that did not show significant correlation were genu and splenium of corpus callosum, which showed the highest MVF_SyMRI_. Meanwhile, only 3 and 4 ROIs showed significant correlation between MVF_MTsat_ and MVF_T1w/T2w_, and MVF_SyMRI_ and MVF_T1w/T2w_, respectively. Both of the 2 subcortical GM ROIs showed significant correlations in all comparisons, with comparison between MVF_MTsat_ and MVF_SyMRI_ showing the highest and strong correlation coefficients. For all the 4 cortical GM ROIs, comparison among MVF_SyMRI_ and MVF_T1w/T2w_ revealed the highest and significant correlations, whereas only 1 ROI (precentral) showed significant correlation between MVF_SyMRI_ and MVF_MTsat_, and no significant correlation was observed between MVF_MTsat_ and MVF_T1w/T2w_.

**Table 2:** Correlation among MVF_MTsat_, MVF_SyMRI_, and MVF_T1w/T2w_ for WM, subcortical GM, cortical GM, and all regions.

	WM	Subcortical GM	Cortical GM	All regions
MVF_MTsat_ vs. MVF_SyMRI_	0.72 [0.69–0.75]	0.78 [0.72–0.82]	0.57 [0.54–0.60]	0.87 [0.86–0.88]
MVF_MTsat_ vs. MVF_T1w/T2w_	0.38 [0.33–0.44]	0.68 [0.60–0.74]	0.54 [0.51–0.57]	0.80 [0.79–0.81]
MVF_SyMRI_ vs. MVF_T1w/T2w_	0.45 [0.40–0.50]	0.69 [0.61–0.75]	0.75 [0.73–0.77]	0.87 [0.86–0.88]

Data are Spearman’s ρ correlation coefficients and 95% confidence intervals.

Note: Correlations were significant for all regions—alone or combined—among these metrics with *p* values < 0.001.

**Table 3:** Correlation among MVF_MTsat_, MVF_SyMRI_, and MVF_T1w/T2w_ for 48 WM ROIs, and MVF_MTsat_, MVF_SyMRI_, and MVF_T1w/T2w_ in each ROI.

		MVF_MTsat_ vs. MVF_SyMRI_	MVF_MTsat_ vs. MVF_T1w/T2w_	MVF_SyMRI_ vs. MVF_T1w/T2w_	MVF_MTsat_ (%)	MVF_SyMRI_ (%)	MVF_T1w/T2w_ (%)
WM	Genu of corpus callosum	0.38 [−0.08–0.70]	0.06 [−0.39–049]	−0.01 [−0.45–0.43]	42.81 ± 2.01	39.36 ± 1.36	35.87 ± 3.57
	Splenium of corpus callosum	0.40 [−0.05–0.72]	−0.05 [−0.48–0.40]	0.15 [−0.31–0.56]	35.49 ± 1.52	39.08 ± 1.47	33.26 ± 3.50
	Anterior limb of internal capsule	0.67*** [0.45–0.81]	0.41* [0.11–0.64]	0.40* [0.10–0.63]	28.88 ± 1.50	33.58 ± 2.08	33.70 ± 3.82
	Posterior limb of internal capsule	0.64*** [0.41–0.79]	0.27 [−0.045–0.54]	0.24 [−0.081–0.51]	28.51 ± 1.21	33.65 ± 1.83	30.67 ± 3.28
	Anterior corona radiata	0.68*** [0.47–0.82]	0.34* [0.034–0.59]	0.44** [0.15–0.66]	36.26 ± 1.42	34.56 ± 1.30	32.84 ± 3.36
	Superior corona radiata	0.57*** [0.32–0.75]	0.23 [−0.092–0.50]	0.37* [0.06–0.61]	32.39 ± 1.26	29.86 ± 1.64	28.59 ± 3.06
	Posterior corona radiata	0.54*** [0.27–0.73]	−0.052 [−0.36–0.26]	0.23 [−0.085–0.51]	30.55 ± 1.13	31.02 ± 1.37	28.80 ± 2.98
	Posterior thalamic radiation	0.65*** [0.43–0.80]	0.10 [−0.22–0.40]	0.099 [−0.22–0.40]	31.87 ± 1.48	36.18 ± 1.34	30.62 ± 3.05
	External capsule	0.66*** [0.43–0.80]	0.31* [0.001–0.57]	0.16 [−0.16–0.45]	27.20 ± 1.17	28.95 ± 1.31	31.21 ± 3.17
	Superior longitudinal fasciculus	0.47** [0.19–0.68]	0.20 [−0.12–0.48]	0.60*** [0.35–0.77]	33.11 ± 1.30	31.36 ± 1.48	29.81 ± 3.18
Subcortical GM	Pallidum	0.72*** [0.53–0.85]	0.53** [0.26–0.72]	0.52** [0.24–0.71]	21.97 ± 1.41	22.34 ± 3.22	35.29 ± 3.88
	Thalamus	0.72*** [0.53–0.84]	0.41** [0.12–0.64]	0.50** [0.22–0.70]	22.60 ± 1.38	20.66 ± 2.26	27.77 ± 3.00
Cortical GM	Precentral	0.41** [0.12–0.64]	0.12 [−0.20–0.42]	0.53** [0.26–0.72]	13.27 ± 1.43	7.99 ± 1.21	19.60 ± 2.19
	Postcentral	0.24 [−0.076–0.51]	−0.10 [−0.40–0.22]	0.59*** [0.34–0.76]	13.22 ± 1.42	8.25 ± 1.19	19.38 ± 2.06
	Heschl	0.20 [−0.12–0.48]	0.098 [−0.22–0.40]	0.35* [0.041–0.60]	14.06 ± 1.93	7.46 ± 1.51	20.05 ± 2.17
	Lingual	0.24 [−0.079–0.51]	0.25 [−0.069–0.52]	0.47** [0.18–0.68]	12.35 ± 1.17	7.10 ± 0.85	20.27 ± 2.08

Data are the Spearman’s ρ correlation coefficients ± 95% confidence intervals or the mean ± standard deviation.

Abbreviations: MVF = myelin volume fraction; MT_sat_ = magnetization transfer saturation; SyMRI = simultaneous tissue relaxometry of R_1_ and R_2_ relaxation rates and proton density; T_1_w/T_2_w = ratio of T_1_-weighted to T_2_-weighted images; WM = white matter; GM = gray matter; ROI = region of interest.

Note: *p < 0.05, **p < 0.01, ***p < 0.001.

### Regression Analysis of MVF_SyMRI_ and MVF_T1w/T2w_ as a Function of MVF_MTsat_

Table 4 shows the values of the intercept and slope with their standard error in each region—alone or combined—for MVF_SyMRI_ and MVF_T1w/T2w_ as a function of MVF_MTsat_. In WM, cortical GM, and all regions combined, significant difference was detected between the slopes of MVF_SyMRI_ and MVF_T1w/T2w_, with that of MVF_SyMRI_ nearer to 1. In subcortical GM, slopes of MVF_SyMRI_ and MVF_T1w/T2w_ did not show statistical significance, and y-intercepts differed significantly with that of MVF_T1w/T2w_ nearer to 0.

**Table 4:** Intercept and slope of MVF_SyMRI_ and MVF_T1w/T2w_ as a function of MVF_MTsat_ for each region—alone or combined.

		Intercept	Slope
WM	MVF_SyMRI_	6.01 ± 0.81	0.81 ± 0.026
	MVF_T1w/T2w_	19.71 ± 0.88	0.36 ± 0.029
Subcortical GM	MVF_SyMRI_	−9.29 ± 1.49	1.23 ± 0.072
	MVF_T1w/T2w_	−0.45 ± 2.32	1.34 ± 0.11
Cortical GM	MVF_SyMRI_	−8.65 ± 0.35	1.19 ± 0.021
	MVF_T1w/T2w_	9.63 ± 0.28	0.71 ± 0.017
All regions	MVF_SyMRI_	−9.93 ± 0.20	1.30 ± 0.0088
	MVF_T1w/T2w_	11.1 ± 0.18	0.64 ± 0.0081

Data are the mean ± standard error.

## Discussion

In this study, we investigated the concurrent validity of SyMRI myelin measurement method by comparing SyMRI with MT_sat_ and T_1_w/T_2_w ratio in WM and GM. As part of the study, we tried to estimate the absolute myelin partial volume in a voxel by these three methods. SyMRI directly estimates MVF of a voxel by bloch simulation. On the other hand, MT_sat_ and T_1_w/T_2_w ratio require calibration to be used as quantitative measures of myelin content. Thus, we calibrated MT_sat_ and T_1_w/T_2_w ratio for their means in the whole WM to be equal to that of MVF_SyMRI_, partly because calibration method does not affect correlation coefficient and contrast between WM and cortical or subcortical GM. In this study, the mean MVF_SyMRI_ in the WM was 30.70%. This corresponds to the previously reported values (around 25–30%) of MVF in WM, investigated by histology^2,44^. This value also corresponds to the results of MVF investigated using SyMRI for WM of cadavers (30.98%)^13^ and normal-appearing WM of MS patients (32.88% and 30.96%)^17,18^. For GM, reports on investigation into MVF by histology are rather scarce and most were performed with optical density using Luxol Fast Blue stain, which could be used only in comparison with the values of other brain microstructures^45^. Previous studies that investigated volume fraction of myelin in the brain showed optical densities of subcortical and cortical GM to be around 49–67% and 9.8–36% that of WM, respectively^13,46^. In our study, MVF_SyMRI_ corresponded to the results of these histological studies in cortical GM better than MVF_MTsat_ and MVF_T1w/T2w_. For subcortical GM, MVF_MTsat_ and MVF_SyMRI_ were comparable and these showed better correspondence to previous histological study than MVF_T1w/T2w_. In terms of WM to GM contrast, we conclude that MVF_SyMRI_ was the best fit to the results of previous histological studies among the metrics investigated in our study.

In our study, we investigated the correlation among three different metrics for myelin content. The aim was to show the concurrent validity of MVF_SyMRI_ by MVF_MTsat_ and MVF_T1W/T2W_. For WM, MVF_SyMRI_ showed strong and higher correlation with MVF_MTsat_ than MVF_T1w/T2w_. In regression analysis, the slope was closer to 1 for MVF_SyMRI_ than MVF_T1w/T2w_ as a function of MVF_MTsat_ in WM. These results are in line with the study by Arshad *et al*.^40^. They investigated the correlation between T_1_w/T_2_w ratio and myelin water fraction in WM, and found that T_1_w/T_2_w ratio poorly correlated with myelin water fraction and correlated more with geometric mean of multi-echo T_2_ relaxation, which had been shown to correlate with axon diameter based on histology, rather than myelin content^47^. Another study also showed poor correlation between T_1_w/T_2_w and myelin water fraction^43^. Therefore, T_1_w/T_2_w ratio may not be a suitable candidate as a measure of myelin in WM. In cortical GM, these three MVF metrics showed moderate to strong correlations to each other, with MVF_SyMRI_ and MVF_T1w/T2w_ showing a higher correlation. However, we cannot determine which is the best measure for estimating myelin content in GM among these three metrics at this moment. Myeloarchitecture is different among cortical areas, and high-resolution T_1_w/T_2_w ratio has been widely used for cortical parcellation, especially in the Human Connectome Project, showing good results^48^. In a future study, comparison of these metrics for the ability of cortical parcellation should be investigated. However, recent histological study showed that T_1_w/T_2_w ratio in the cerebral cortex correlated well with dendrites, but not with myelin, even though the sample size was small (9 MS patients)^42^. There is a possibility that T_1_w/T_2_w ratio does not reflect actual myelin content in the brain. All regions in aggregate showed strong correlation coefficients in all comparisons (i.e. MVF_MTsat_ vs. MVF_SyMRI_, MVF_MTsat_ vs. MVF_T1w/T2w_, and MVF_SyMRI_ vs. MVF_T1w/T2w_). This may be because subgroups with different microstructures were included in the analysis.

When we analyzed individual structures representative of WM, subcortical GM, and cortical GM, the correlation coefficients showed similar tendency to those shown for each segment as a whole. Of note, only genu and splenium of corpus callosum out of the 10 WM ROIs did not show significant correlation between MVF_MTsat_ and MVF_SyMRI_, with these showing the highest MVF_SyMRI_. This may be because SyMRI does not assume nonphysiological MVF higher than 40%^12^, and disagreement may have occurred between SyMRI and MT_sat_ with high values.

Determination of the precise relationship between MRI measures of myelin and actual MVF is especially important for calculating the g-ratio, which is the ratio of the inner and the outer diameter of a myelinated nerve fiber^49^. Calculation of the g-ratio by MRI can be performed with myelin imaging in combination with diffusion MRI, such as diffusion tensor imaging (DTI) and neurite orientation dispersion and density imaging (NODDI)^49,50^. Because diffusion MRI alone is not sufficient to estimate axon volume fraction^49^, precise measurement of myelin is necessary for correct g-ratio calculation. Furthermore, g-ratio could complement MVF measurements in understanding tissue microstructure, because MVF only cannot differentiate partial demyelination of neuronal fibers from loss of axons, with the remaining axons fully myelinated. Thus, g-ratio can provide a more complete picture of the microstructure, which is important for understanding plasticity of the normal brain^51^ and may also be important for the care of patients with MS in choosing immunotherapy or remyelination therapy^25^. Because we could not perform histological measurements of actual myelin content in this study, we calibrated MT_sat_ and T_1_w/T_2_w ratio to MVF_SyMRI_. Even though we assumed zero-intercept upon calibration of MVF_MTsat_ and MVF_T1w/T2w_ to MVF_SyMRI_, we detected a non-zero intercept when linear regression was performed. This means that at least two of these MVF metrics are not perfectly specific to myelin content in the brain. Although it may be expected that MT_sat_ is also sensitive to macromolecules other than myelin, the specificity of our MVF metrics to actual myelin content should be investigated more precisely in future histological studies. We should also be aware that scaling factors depend on the acquisition protocol and post-processing, and should be carefully determined for each investigation^25^.

Rapid relaxation of myelin water cannot be directly measured by the SyMRI sequence, but the presence of MVF can be inferred by its effect of magnetization exchange with the slower cellular relaxation, as well as the decrease in observed PD. This is an indirect measurement and may have some limitations when compared with a more direct approach, such as myelin water fraction, which estimates T_2_ distribution of water including myelin water by fitting multi-exponential T_2_ decay^22^ and has been shown to correlate well with histological myelin content in patients with MS^52^. However, for clinical use, the robustness and easy implementation may be more important. SyMRI myelin measurement has been shown to have good repeatability, which is important for longitudinal studies^14^. In addition to myelin measurements, any contrast-weighted image can also be generated by SyMRI^53^, thus obviating the need for further conventional scans.

There are several limitations in this study. First, the resolutions of the images were different between MVF_SyMRI_ or T_1_w/T_2_w ratio (2D acquisition) and MT_sat_ (3D acquisition). Even though the difference in resolution could introduce deviation in the quantification, this would have been offset by a large number of ROIs used in this study. However, the analyses of 2D and 3D images by consistent methods was a challenge in our study. Rather than co-registering these images, we registered ROIs in template space to 2D or 3D space for each subject. Co-registration may cause some mis-registration, which will result in inappropriate comparison of voxels derived from different tissues. When we applied the ROIs to each MVF map, we used partial volume maps of GM, WM, or both, with thresholding, to minimize partial volume effects. Second, T_1_-weighted images for T_1_w/T_2_w ratio were acquired by a spin-echo sequence, even though mostly gradient-echo sequences have been used for calculating T_1_w/T_2_w ratio^33,35,36,40,48^. Because T_1_w/T_2_w ratio is a semi-quantitative value, different acquisitions may introduce different contrasts. However, T_1_w/T_2_w ratio has been shown to give very similar overall results when acquired on different scanners with different sequences and different field strengths^33,35^. Third, the myelin measurement methods investigated in this study may show variable behaviors in diseased brains from healthy brains, not only due to demyelination but also due to edema, inflammation, iron accumulation, or atrophy. This should be investigated in future studies. For example, MTR seems to correlate with not only myelin but also with change in water content caused by inflammation or edema in patients with MS^54^. Even though we assumed a linear relationship for calibration of MVF values, this assumption may not hold true in diseased brains.

In summary, we compared MT_sat_, MVF_SyMRI_, and T_1_w/T_2_w ratio as quantitative measures of myelin in the brain. We calibrated MT_sat_ and T_1_w/T_2_w in WM to be equal to MVF_SyMRI_ in WM (MVF_MTSat_ and MVF_T1w/T2w_). Correlation of these metrics in WM was strong and higher between MVF_MTsat_ and MVF_SyMRI_ than between MVF_T1w/T2w_ and MVF_MTsat_ or MVF_SyMRI_, indicating that MVF_MTsat_ and MVF_SyMRI_ are similarly suited to measure myelin in the WM, whereas MVF_T1w/T2w_ may be less optimal. In GM, moderate to strong correlation was observed among these metrics. However, further studies performing cortical parcellation using these measures or investigating the correlation between each MVF metric and histology should be conducted before concluding which is the best measure for estimating myelin content in GM.

## Materials and Methods

### Study Participants

Twenty healthy volunteers (9 male and 11 female, mean age 55.3 years, age range 25–71 years) were included in this study. These subjects were screened by a questionnaire for neurological or psychological symptoms, or history of neurologic diseases. Acquired images were also screened for moderate-to-severe WM ischemic lesions (Fazekas grade 2 or more^55^), asymptomatic cerebral infarction, or regional brain atrophy.

### Ethical issue

All data from the patients were obtained in accordance with the 2013 revised Helsinki Declaration of 1964. We provided participants with detailed information, and written informed consent was obtained from all participants. The Ethical Committee of Juntendo University Hospital approved the study.

### MRI Acquisition Protocol for SyMRI

All subjects were scanned on a single 3T MRI scanner (MAGNETOM Prisma, Siemens Healthcare, Erlangen, Germany) using a 64-channel head coil. QRAPMASTER (an acronym derived from ‘quantification of relaxation times and proton density by multi-echo acquisition of a saturation-recovery by using turbo spin-echo readout’ for simultaneous tissue relaxometry) was performed for all subjects. QRAPMASTER is a two-dimensional (2D) axial multi-slice, multi-echo, and multi-saturation delay saturation-recovery turbo spin-echo acquisition method with which images are collected with different combinations of echo times (TEs) and saturation delay times. In our institution, combinations of 2 TEs and 4 delay times were used to make a matrix of 8 complex images that were then used to quantify longitudinal R_1_ relaxation and transverse R_2_ relaxation rates and PD by using SyMRI software 8.0 (SyntheticMR, Linköping, Sweden). The TEs were 22 and 99 ms, and the delay times were 170, 620, 1970, and 4220 ms. The repetition time (TR) was 4250 ms. The other parameters used for QRAPMASTER were as follows: field of view (FOV) 230 × 186 mm; matrix 320 × 260; echo-train length 10; bandwidth 150 Hz/pixel; parallel imaging acceleration factor 2; slice thickness/gap 4.0 mm/1.0 mm; 30 sections; and acquisition time 5 min 8 sec.

### Processing of SyMRI Data

Based on the R_1_, R_2_, and PD values acquired by QRAPMASTER, myelin volume fraction (MVF_SyMRI_) was also calculated automatically on SyMRI software. This model for myelin measurement hypothesizes 4 compartments in the brain: myelin, cellular, free water, and excess parenchymal water partial volumes^12^. The model assumes that the relaxation behavior of each compartment contributes to the effective relaxation behavior of an acquisition voxel. The R_1_, R_2_, and PD values of free water and excess parenchymal water partial volumes were fixed to those of cerebrospinal fluid (CSF) (R_1_, 0.24 sec^−1^; R_2_, 0.87 sec^−1^; PD, 100%)^8^. The R_2_ of myelin partial volume was fixed to the literature value of 77 sec^−1^ ^56^. Optimization of other model parameters were done by performing simulation by running Bloch equations for observable R_1_, R_2_, and PD properties in a spatially normalized and averaged brain from a group of healthy subjects^12^. In this model, the magnetization exchange rates between partial volume compartments are also considered. A lookup grid was made in R_1_-R_2_-PD space for all possible distributions (ranging from 0% to 100%) of the four partial volumes. The measured R_1_, R_2_, and PD values were projected onto the lookup grid, for estimating the MVF_SyMRI_ in each voxel. Although other methods for myelin imaging require scaling factors to estimate MVF from measured macromolecular pool size or myelin water fraction, assuming linear proportionality^2^, we omitted this procedure because MVF_SyMRI_ directly estimates the volume fraction of myelin in a voxel^12^.

### Processing of T1w/T2w ratio

Synthetic T_1_w and T_2_w images were produced from QRAPMASTER data. Parameters used for T_1_w images were: TR 500 ms; and TE 10 ms. Parameters used for T_2_w images were: TR 4500 ms; and TE 100 ms. These T_1_w and T_2_w images were intrinsically aligned. Synthetic T_1_w and T_2_w images were skull-stripped using the intracranial mask generated by SyMRI software^57^. In conventional MRI, imperfection of B_1_ field affects T_1_w and T_2_w images, generating intensity non-uniformity in these images. It has been proposed that this non-uniformity should be corrected before the ratio of these images is calculated, because a ratio does not adequately cancel the intensity non-uniformity^35^. The QRAPMASTER sequence acquires the B_1_ field map and the acquired quantitative data are automatically corrected for local B_1_ field when processed by SyMRI software^9^. Because T_1_w and T_2_w images are non-quantitative, the intensity scaling may vary among different individuals, sequences, or scanners. To minimize the effect of intensity scaling, we applied an external linear calibration to these contrast-weighted images as proposed by Ganzetti *et al*.^35^, which would provide a more consistent range of T_1_w and T_2_w intensities even across different datasets. Two masks of anatomical structures external to the brain—one with high T_1_w signal intensity and low T_2_w signal intensity (temporalis muscle) and the other with opposite properties (eye)—were used for calibration. These regions were defined in the MNI152 space using the ICBM152 template images (http://www.bic.mni.mcgill.ca/ServicesAtlases/ICBM152NLin2009) and then warped to each subject’s space using the registration matrix described below in the ROI Analysis section. Distribution peaks (modes) of intensity values were recorded for these regions of interest (ROIs) in each subject. In ICBM152 template images, we recorded the modes as reference values for the eyes as following: 28.2 for T_1_w images and 99.9 for T_2_w images. For the temporalis muscle, the values were: 58.6 for T_1_w images and 21.1 for T_2_w images. The linear scaling of either T_1_w or T_2_w images was performed using the following equation^35^:

1 $${I}_{C}=[\frac{{E}_{R}-{M}_{R}}{{E}_{S}-{M}_{S}}]\times I+[\frac{{E}_{S}{M}_{R}-{E}_{R}{M}_{S}}{{E}_{S}-{M}_{S}}]$$

where I and I_C_ represent the images before and after calibration. E_S_ and M_S_ are the mode intensity values of each subject’s eye and muscle masks, respectively, and E_R_ and M_R_ show the reference values in template images of eye and muscle masks, respectively. After calibrating the T_1_w and T_2_w images, their ratio was calculated to produce the T_1_w/T_2_w ratio images.

### Acquisition and Processing of MT_sat_

Three three-dimensional (3D) multi-echo fast low-angle shot (FLASH) sequences were performed with predominant T_1_-, PD-, and MT-weighting for all subjects. For T_1_w images, TR/excitation flip angle α = 10 ms/13° were used; for PD- and MT-weighted images, 24 ms/4° were used. For MT-weighted images, excitation was preceded by an off-resonance Gaussian-shaped RF pulse (frequency offset from water resonance 1.2 kHz, pulse duration 9.984 ms, and nominal flip angle 500°). For the other parameters, the following was used: slice thickness 1.8 mm; 104 slices; FOV 224 × 224 mm; matrix 128 × 128, parallel imaging using GRAPPA factor 2 in phase-encoding direction; 7/8 partial Fourier acquisition in the partition direction; bandwidth 260 Hz/pixel; and total acquisition time 6 min 25 sec.

These three images were used to calculate the MT_sat_ index^31^. First, the apparent longitudinal relaxation rate R_1app_ was calculated as follows:

2 $${R}_{1{\rm{app}}}=\frac{1}{2}\frac{{S}_{{\rm{T}}1}{\alpha }_{{\rm{T}}1}/{{\rm{TR}}}_{{\rm{T}}1}-{S}_{{\rm{PD}}}{\alpha }_{{\rm{PD}}}/{{\rm{TR}}}_{{\rm{PD}}}}{{S}_{{\rm{PD}}}/{\alpha }_{{\rm{PD}}}-{S}_{{\rm{T}}1}/{\alpha }_{{\rm{T}}1}}$$

where S_T1_ and S_PD_ denote signal intensities of T_1_w and PD-weighted images, respectively; TR_T1_ and TR_PD_ denote TR of T_1_w and PD-weighted images, respectively; and α_T1_ and α_PD_ denote excitation flip angles of T_1_w and PD-weighted images, respectively.

Secondly, the apparent signal amplitude A_app_ was calculated as follows:

3 $${A}_{{\rm{app}}}={S}_{{\rm{PD}}}{S}_{{\rm{T}}1}\frac{{{\rm{TR}}}_{{\rm{PD}}}{\alpha }_{{\rm{T}}1}/{\alpha }_{{\rm{PD}}}-{{\rm{TR}}}_{{\rm{T}}1}{\alpha }_{{\rm{PD}}}/{\alpha }_{{\rm{T}}1}}{{S}_{{\rm{T}}1}{{\rm{TR}}}_{{\rm{PD}}}{\alpha }_{{\rm{T}}1}-{S}_{{\rm{PD}}}{{\rm{TR}}}_{{\rm{T}}1}{\alpha }_{{\rm{PD}}}}$$

Thirdly, the apparent MT saturation δ_app_ was calculated as follows:

4 $${\delta }_{{\rm{app}}}=({A}_{{\rm{app}}}{\alpha }_{{\rm{MT}}}/{S}_{{\rm{MT}}}-1){R}_{1{\rm{app}}}{{\rm{TR}}}_{{\rm{MT}}}-{\alpha }_{{\rm{MT}}}^{2}/2$$

where S_MT_, TR_MT_, and α_MT_ denote signal intensity, TR, and excitation flip angle of MT-weighted image, respectively.

The apparent MT saturation is inherently robust against differences in relaxation rates and inhomogeneities of RF transmit and receive field compared with conventional MTR imaging^31,58^. Furthermore, we also corrected for small residual higher-order dependencies of the MT saturation on the local RF transmit field to further improve spatial uniformity, as suggested by Weiskpof *et al*.^59^:

5 $$M{T}_{{\rm{sat}}}=\frac{{\delta }_{{\rm{app}}}(1-0.4)}{1-0.4R{F}_{{\rm{local}}}}$$

where RF_local_ is the relative local flip angle α compared to the nominal flip angle. RF_local_ was calculated by dual-angle method^60^. For this method, two additional B1 maps using echo-planar imaging with nominal 10° and 20° flip angles were acquired in short acquisition time (around 10 seconds each). The first image was acquired after excitation with a flip angle α and had a magnitude proportional to sin(α). The second image was acquired after excitation with a flip angle 2α and had a magnitude proportional to sin(2α). The ratio of the two acquisitions was formed giving:

6 $$\frac{\sin \,\alpha }{\sin \,2\alpha }=\frac{1}{2\,\cos \,\alpha }$$

from which the local flip angle α was calculated.

### ROI Analysis

We used Johns Hopkins University (JHU) ICBM-DTI-81 WM labels atlas^61,62^ and the automated anatomical labeling (AAL) atlas^63,64^ to define WM and GM ROIs, respectively. The JHU ICBM-DTI-81 WM labels atlas comprised 48 WM ROIs; AAL comprised 116 ROIs including 12 subcortical GM ROIs. Even though MVF_SyMRI_ and T_1_w/T_2_w ratio were in an identical space with the same resolution and slice thickness, MT_sat_ had a different resolution and slice thickness. To ensure that ROIs were placed in the same anatomical position in these different spaces, we warped the above ROIs to each metric map.

**Figure 2:**
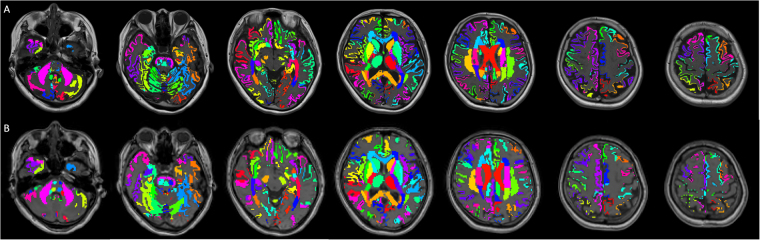
Examples of ROI placement are shown for a 56-year-old female subject. (**A** and **B**) show transformed ROIs overlaid on 2D synthetic and 3D T_1_-weighted images in the same subject, respectively. Transformed ROIs for cortical GM and WM were masked by GM and WM partial volume maps thresholded at 0.95, respectively. For subcortical GM ROIs, GM plus WM partial volume maps thresholded at 0.95 were used for masking. For analysis, ROIs transformed to 2D synthetic T_1_-weighted images were applied to MVF_SyMRI_ and T_1_w/T_2_w ratio, and ROIs transformed to 3D T_1_-weighted images were applied to MT_sat_.

For generating the warp field to convert ROIs in the template space to each subject’s space, we first used the FMRIB Software Library (FSL) linear and nonlinear image registration tool (FLIRT and FNIRT)^65,66^ to register synthetic T_1_w and 3D T_1_w images to the MNI152 template. The generated warp fields were saved and inverted so they could be applied to all ROIs, including the eye and temporalis muscle masks. Next, to remove the partial volume effects from other tissues, we segmented synthetic T_1_w and 3D T_1_w images into WM, GM, and CSF using FMRIB’s Automated Segmentation Tool (FAST)^67^. These segmented images of WM and GM were used as masks and applied to MVF_SyMRI_, T_1_w/T_2_w ratio, and MT_sat_. These tissue masks were thresholded at 0.95 to make sure that the masks contained WM or GM with a probability of 0.95 or higher. WM plus GM tissue masks were also made and thresholded at 0.95. For MVF_SyMRI_ and T_1_w/T_2_w ratio, we used tissue masks based on the synthetic T_1_w images; for MT_sat_, we used tissue masks made from 3D T_1_-weighted images. For applying the ROIs from the JHU ICBM-DTI-81 WM labels atlas, we used MVF_SyMRI_, T_1_w/T_2_w ratio, and MT_sat_ masked by WM tissue masks. For applying the ROIs from the AAL atlas to cortical GM, we used MVF_SyMRI_, T_1_w/T_2_w ratio, and MT_sat_ masked by GM tissue masks. For applying the ROIs from the AAL atlas to subcortical GM (e.g., thalamus), we used MVF_SyMRI_, T_1_w/T_2_w ratio, and MT_sat_ masked by GM plus WM tissue masks, because many parts of subcortical GM were segmented as WM by FAST. After warping, all ROIs were inspected for gross registration errors. Upon ROI analysis, the mean values were recorded for further analysis. Examples of ROI placement are shown in Fig. 2.

### Calibration of MVF

**Figure 3:**
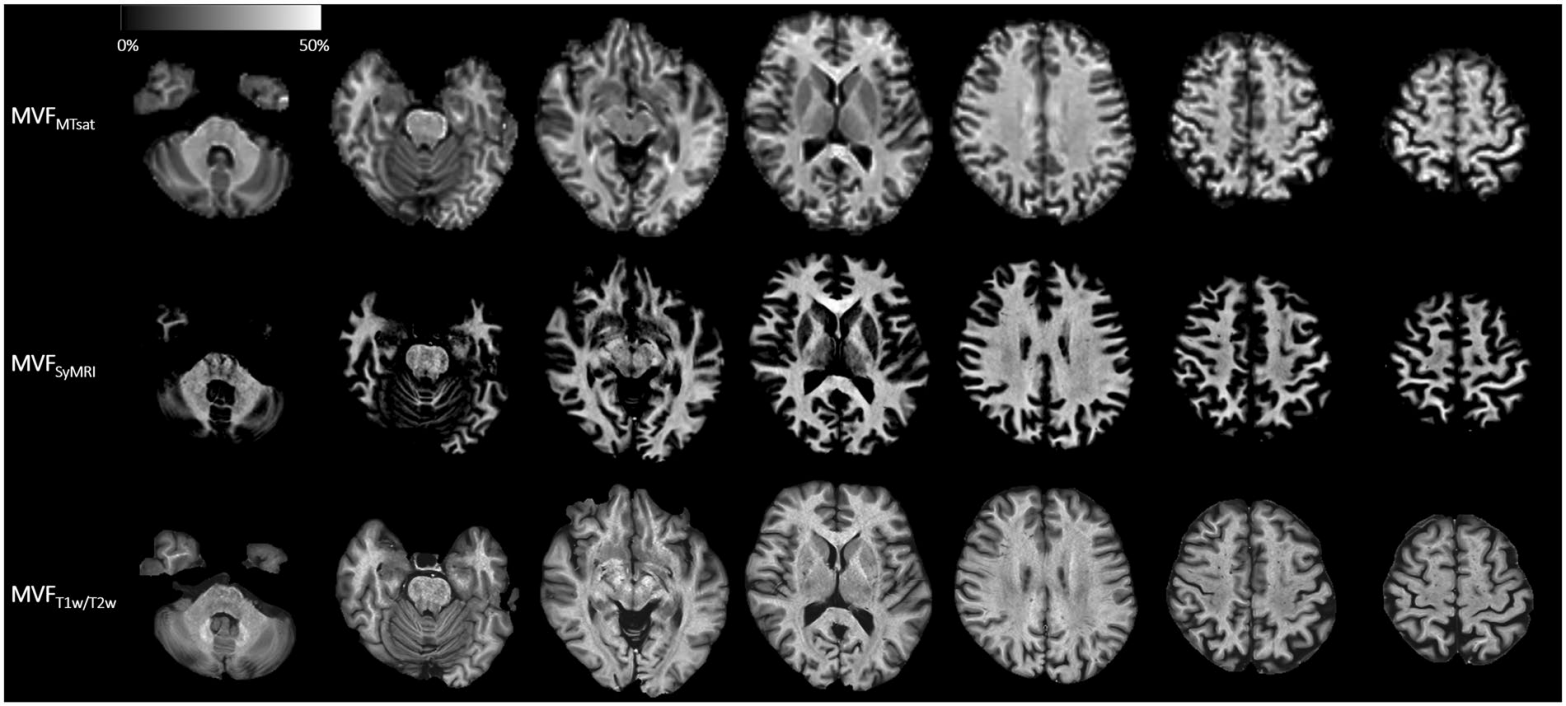
MVF_MTsat_, MVF_SyMRI_, and MVF_T1w/T2w_ maps of the same subject as Fig. 2 are shown. Because MVF_MTsat_ and MVF_T1w/T2w_ were calibrated for their mean in the whole WM to be equal to the mean MVF_SyMRI_, these maps look similar to each other in WM. On the contrary, these maps show great variability in GM, with MVF_SyMRI_ showing the highest contrast between WM and GM, and MVF_T1w/T2w_ showing the lowest contrast between WM and GM.

Even though SyMRI directly estimates MVF of a voxel, MT_sat_ and T_1_w/T_2_w cannot be used as quantitative myelin markers as they are. For calibration of MT_sat_ and T_1_w/T_2_w ratio to be used for quantifying myelin in the brain, we assumed a linear relationship between MVF_SyMRI_, MT_sat_, T_1_w/T_2_w ratio, and actual myelin content, as described previously for MT_sat_^68^. In the brain, not only myelin, but also other microstructures contribute to the values of MT_sat_ and T_1_w/T_2_w ratio. However, if we assume a linear relationship between MT_sat_ or T_1_w/T_2_w ratio and actual myelin content, MT_sat_ or T_1_w/T_2_w ratio would also correlate linearly with non-myelin microstructures. Hence, the intercept of the regression line of actual myelin on MT_sat_ or T_1_w/T_2_w would be near to zero. Since several studies have calibrated scaling factors of myelin sensitive metrics by healthy WM^25,49,68^, we also decided to calibrate MT_sat_ and T_1_w/T_2_w ratio by values of WM. We determined the scaling factors of T_1_w/T_2_w ratio and MT_sat_ by making the means of these values in all the 48 WM ROIs equal to the mean MVF_SyMRI_. We denoted calibrated MT_sat_ and T_1_w/T_2_w ratio as MVF_MTsat_ and MVF_T1w/T2w_, respectively. Maps of MVF_MTsat_, MVF_SyMRI_, and MVF_T1w/T2w_ are shown in Fig. 3. After calibration, we performed ROI analysis again for MVF_T1w/T2w_ and MVF_MTsat_ as described in the previous section and mean values were recorded.

### Statistical analysis

For MVF values, normality was tested with the Shapiro-Wilk test. All of the datasets were not normally distributed; therefore, we used the Steel-Dwass test, which is a nonparametric test for multiple comparisons, to compare the contrast among WM and cortical GM, and WM and subcortical GM for the three MVF metrics, and used Spearman’s rank order correlation coefficient to investigate the correlation among MVF metrics for WM, subcortical GM, and cortical GM. Spearman’s ρ correlation coefficients were classified by using the following definitions: 0–0.30, very weak; 0.30–0.50, weak; 0.50–0.70, moderate; 0.70–0.90, strong; and 0.90–1.00, very strong^69^. Comparison of correlation coefficients among MVF_MTsat_ vs. MVF_SyMRI_, MVF_MTsat_ vs. MVF_T1w/T2w_, and MVF_SyMRI_ vs. MVF_T1w/T2w_ were performed in WM, subcortical GM, and cortical GM. This was performed with the Z test for the equality of the two correlations after Fisher r-to-Z transformation^70^. In addition to analyzing each segment as a whole, we also performed correlation analysis in individual structures representative of WM (genu of corpus callosum, splenium of corpus callosum, anterior limb of internal capsule, posterior limb of internal capsule, anterior corona radiata, superior corona radiata, posterior corona radiata, posterior thalamic radiation, external capsule, and superior longitudinal fasciculus), subcortical GM (pallidum and thalamus), and cortical GM (precentral, postcentral, Heschl, and lingual). Other than corpus callosum, we used bilateral regions aggregately in the analysis. Simple linear regression analysis was performed on the MVF_SyMRI_ and MVF_T1w/T2w_ as a function of MVF_MTsat_. The regression lines for MVF_SyMRI_ and MVF_T1w/T2w_ were compared by analysis of covariance to determine if they were significantly different from each other in WM, subcortical GM, cortical GM, and all regions combined. All statistical analyses were performed with the software package R, version 3.2.1 (http://www.r-project.org/). A 2-sided *p* value < 0.05 was considered significant.

### Data availabillity

The datasets generated during and/or analyzed during the current study are available from the corresponding author on reasonable request.
